# The Effect of Chalazion Excision on Corneal Aberrometric and Densitometric Values

**DOI:** 10.14744/bej.2021.62533

**Published:** 2021-09-27

**Authors:** Hasan Oncul, Yusuf Yildirim, Mehtap Caglayan, Umut Dag, Mehmet Fuat Alakus

**Affiliations:** Department of Ophthalmology, University of Health Sciences Gazi Yasargil Training and Research Hospital, Diyarbakir, Turkey

**Keywords:** Chalazion, corneal densitometry, pentacam HR

## Abstract

**Objectives:**

The aim of this study was to investigate the effect of chalazion excision on corneal aberrations and corneal densitometry.

**Methods:**

Thirty-six patients with a chalazion in 1 eyelid and 40 healthy subjects were included in the study. Corneal aberration parameters of total root mean square (RMS), RMS high-order aberration (HOA), horizontal trefoil, oblique trefoil, horizontal coma, vertical coma, and spherical aberration values were measured using Scheimpflug corneal topography (Pentacam HR; Oculus Optikgeräte GmbH, Wetzler, Germany). Corneal densitometry values measured from 4 regions of the cornea (0–2, 2–6, 6–10, and 10–12 mm) and 4 corneal depths (anterior, central, posterior, and total) were recorded. Preoperative measurements of the patients (Group 1), postoperative first-month measurements (Group 2), and control group (Group 3) measurements were compared.

**Results:**

The total RMS measurement was 1.64±0.48 μm in Group 1, 1.35±0.32 μm in Group 2, and 1.17±0.38 μm in Group 3 (Group 1–2: p=0.007, Group 1–3: p<0.001, Group 2-3: p=0.173). The mean spherical aberration value was 0.183±0.057 μm in Group 1, 0.157±0.048 μm in Group 2, and 0.144±0.050 μm in Group 3 (Group 1–2: p=0.104, Group 1-3: p=0.004, Group 2–3: p=0.781). The total corneal densitometric measurement was 15.95±1.80 gray scale units (GSU) in Group 1, 14.76±1.76 GSU in Group 2, and 14.33±1.49 GSU in Group 3 (Group 1–2: p=0.01, Group 1–3: p<0.001, Group 2–3: p=0.804).

**Conclusion:**

It was observed that some corneal aberration and corneal densitometry values were higher in patients with a chalazion compared with those of healthy individuals, and there was a decrease in corneal aberration and densitometry values after surgical excision.

## Introduction

A chalazion is a lipogranulomatous inflammation that occurs due to blockage of the secretory ducts of the meibomian glands in the eyelids and may affect all ages of people (1). Causes of meibomian gland dysfunction, such as chronic blepharitis, rosacea, and seborrheic dermatitis, are risk factors for the development of chalazion (2). Ocular disorders such as cosmetic disturbances in the eyelids, visual disturbances, foreign body sensation, and ptosis may also occur (1).

Corneal transparency is considered as an indicator of corneal health and may vary with many factors that cause corneal and endothelial dysfunction (3). Corneal transparency, which is evaluated subjectively by slit-lamp biomicroscopy in an ophthalmic examination, has recently been evaluated quickly and objectively by utilizing the ability of light to reflect back from the corneal layers. Pentacam HR (Oculus, Wetzlar, Germany) is a non-invasive imaging method that can obtain up to 50 anterior segment cross-sectional images within 2 s using the Scheimpflug principle. With added software, it measures corneal aberration and densitometry changes so that the effects of eye and systemic diseases on the cornea can be examined in more detail (4). Densitometry maps of the cornea are obtained by measuring the reflected rays from the corneal epithelium, stroma, and endothelium with the Scheimpflug method. These analyses, in which the scattering of the reflected rays in the optical axis is measured, can be affected by the tissue properties and structure in the path of light, such as the anatomical arrangement of the collagen fibers in the stroma and keratocytes and the extracellular matrix organization (3). Furthermore, this technique allows the measurement of corneal aberrations through optical wavefront analysis (4).

A chalazion is an inflammatory mass that can resorb spontaneously or with medical treatment. Due to mechanical compression on the cornea and limbus, distortion may develop in the cornea and may cause some topographic changes in the cornea (1). In addition to the mechanical compression effect of the chalazion, the existing inflammatory process may also affect the cornea. These changes may cause a change in corneal transparency and ultimately in optical quality. In this study, pre-operative aberrometric and densitometric values were measured in chalazion patients, and these values were compared with a healthy control group and in the post-operative period. To the best of our knowledge, there is no study in the literature evaluating the densitometric properties of the cornea before and after chalazion surgery.

## Methods

This study was conducted in Diyarbakır Gazi Yaşargil Training and Research Hospital between January and October 2020. Ethics committee approval was obtained from the mentioned hospital for the study. Written informed consent forms were obtained from the patients, and the principles of the Helsinki Declaration were followed in the study.

Patients with treatment-resistant chalazion in a single eyelid for at least 1 month were included in the study. The control group consisted of healthy individuals who did not have any ocular and/or systemic disease that could affect corneal measurements. Patients with a history of ocular surgery, a history of ocular trauma, who wore contact lenses, who displayed the presence of a corneal scar, anterior segment, lens, vitreous and macular pathology, and those with systemic diseases likely to cause changes in corneal densitometry were excluded from the study. A detailed examination, including visual examination with Snellen chart (20 feet), best-corrected visual acuity, tonometry, slit-lamp biomicroscopic examination, and dilated fundus examination, was performed in all patients. The presence of dry eye and corneal punctate staining was investigated, as it may affect the corneal densitometry measurement of all participants. The Schirmer I test (without local anesthesia) was applied to detect the presence of dry eye (Color Bar, Eagle Vision, Memphis, TN). After 5 min, wetting less than 5 mm was accepted as severe dry eye; 5–10 mm as mild dry eye; and wetness ≥10 mm was considered normal. The presence of punctate epitheliopathy was evaluated by corneal staining using a fluorescein strip (Haag-Streit, Köniz, Switzerland). Patients with chalazion with a Schirmer I test greater than 10 mm and without punctate style epithelial staining on the cornea were included in the present study. Topographic, aberrometric, and densitometric measurements of the cornea were made with Pentacam HR (Oculus GmbH, Wetzlar, HE, Germany). Measurements were made preoperatively and in the post-operative 1^st^ month. Pre-operative chalazion patients were categorized as Group 1, post-operative chalazion patients as Group 2, and healthy controls as Group 3. Furthermore, corneal measurements were made according to site of chalazion (upper eyelid/lower eyelid).

Measurements made with Pentacam HR were performed by the same experienced physician (X.X) in a dark room (without a window) in the drafting room with standard lighting (4 lux) during the same time of day (2:00–4:00 p.m.), without dilating the pupil. Patients whose extraction quality was not approved by the device were not included in the study. The average of values obtained from two consecutive shots was entered into the database. For corneal densitometry, the random density units (gray scale unit) of the rays scattered back from the cornea were expressed with results ranging from a minimum of 0 (maximum transparency) to a maximum of 100 (minimum transparency-total corneal opacity) (5). To obtain corneal densitometry data, the 12 mm diameter area of the cornea was divided into four concentric zones: 0–2, 2–6, 6–10, and 10–12 mm. Cornea depth was obtained from four different zones: The anterior surface (120 μm), the posterior (60 μm), the stromal layer in between, the central part, and the whole cornea layer. To evaluate corneal aberrations, wavefront measurements were made with Pentacam HR from the 6 mm zone so that the pupil size did not affect the measurements. With Zernike analyses, total root mean square (RMS), RMS high-order aberration (HOA), trefoil 0° (horizontal), trefoil 30° (oblique), coma 0° (horizontal), coma 90° (vertical), and spherical aberration values were recorded.

### Surgical Method

Local anesthesia was provided with 2% lidocaine. The lesion was localized with the help of chalazion forceps, and the stability of the eyelid and lesion was achieved by reversing the eyelid. The chalazion mass was reached with a vertical transconjunctival incision with the 11^th^ scalpel. The mass content and the chalazion capsule were completely curetted with the chalazion curette, and the wound was left for primary healing without suturing. Printed closure was performed for 1 day, and topical antibiotic steroid drops and antibiotic pomade were prescribed.

### Statistical Analysis

The Statistical Package for the Social Sciences (SPSS) 20.0 software for Windows (SPSS Inc., Chicago, Illinois, USA) was used to analyze the outcomes. The compatibility of the data with normal distribution was checked with the Kolmogorov–Smirnov test. Quantitative variables were reported as mean±standard deviation. An independent t-test was used to compare categorical variables between the sides of chalazion. A one-way analysis of variance (ANOVA) was used to compare the studied parameters of the three groups. The Bonferroni post hoc test was used to determine differences between the groups. The Bonferroni correction for post hoc analysis in ANOVA was performed. Furthermore, p<0.05/3 =0.016 was considered statistically significant in the Bonferroni pairwise tests. For all other comparisons, p<0.05 was considered statistically significant.

## Results

Thirty-six patients with chalazion in one eye and 40 healthy controls were included in this study. Whereas the male/female ratio was 11/25 in patients with chalazion, it was 14/25 in the control group. Furthermore, the mean age of patients with chalazion was 24.9±11.3 years, and it was 23.6±7.8 years in the control group. There was no statistical difference between the groups in terms of age and gender (p>0.05).

There was chalazion in the right eye of 14 patients and in the left eye of 22 patients. The lesion was in the upper eyelid in 20 patients and in the lower eyelid in 16 patients. The Schirmer I test revealed results of 16.7±2.7 mm in Group 1, 17.7±2.7 mm in Group 2, and 20.5±3.1 mm in Group 3. Furthermore, the difference between the groups was statistically significant (Groups 1–2: p=0.347, Groups 1–3: p≤0.001, and Groups 2–3: p≤0.001).

Total RMS measurements were 1.64±0.48 μm in Group 1, 1.35±0.32 μm in Group 2, and 1.17±0.38 μm in Group 3 (Groups 1–2: p=0.007, Groups 1–3: p≤0.001, and Groups 2–3: p=0.173). Spherical aberration values were measured as 0.183±0.057 μm in Group 1, 0.157±0.048 μm in Group 2, and 0.144±0.050 μm in Group 3 (Groups 1–2: p=0.104, Groups 1–3: p=0.004, and Groups 2–3: p=0.781). Whereas the difference in trefoil horizontal and coma vertical aberrations between the groups was significant (p<0.001 and p=0.011, respectively), there was no significant difference between trefoil oblique and coma horizontal aberrations (p=0.024 and p=0.391, respectively) (Table 1).

**Table 1.** The comparison of corneal aberration measurements of preoperative chalazion, postoperative chalazion, and the healthy control group

	**Group 1**	**Group 2**	**Group 3**	p*	**p^†^**
	**(Preoperative)**	**(Postoperative)**	**(Control)**		
	**(n=36)**	**(n=36)**	**(n=40)**		
Total RMS (μm)	1.64±0.48	1.35±0.32	1.17±0.38	**<0.001**	**1-2: 0.007**
					**1-3: <0.001**
					**2-3: 0.173**
Spherical	0.183±0.057	0.157±0.048	0.144±0.05	**0.005**	**1-2: 0.104**
Aberration (μm)					**1-3: 0.004**
					**2-3: 0.781**
Trefoil	0.060±0.112	0.121±0.102	-0.031±0.067	**<0.001**	**1-2: 0.291**
Horizontal (μm)					**1-3: <0.001** 2-3: 0.051
Trefoil	-0.006±0.146	-0.052±0.119	-0.083±0.095	**0.024**	**1-2:0.019**
Oblique (μm)					1-3: 0.345
					2-3: 0.762
Coma	0.484±0.146	0.413±0.166	0.001±0.172	0.391	
Horizontal (μm)					
Coma Vertical (μm)	0.720±0.199	-0.063±0.200	-0.004±0.163	**0.011**	**1-2: 0.009**
					1-3: 0.245
					2-3: 0.520

RMS: Root mean square μm: micron meters. Results are denoted as mean±standard deviation. *: One-way analysis of variance (ANOVA); p<0.05 statistically significant. ^†^: Bonferroni Post-hoc test; p<0.016 statistically significant. (Bold value indicates statistically significant).

**Figure 1.**
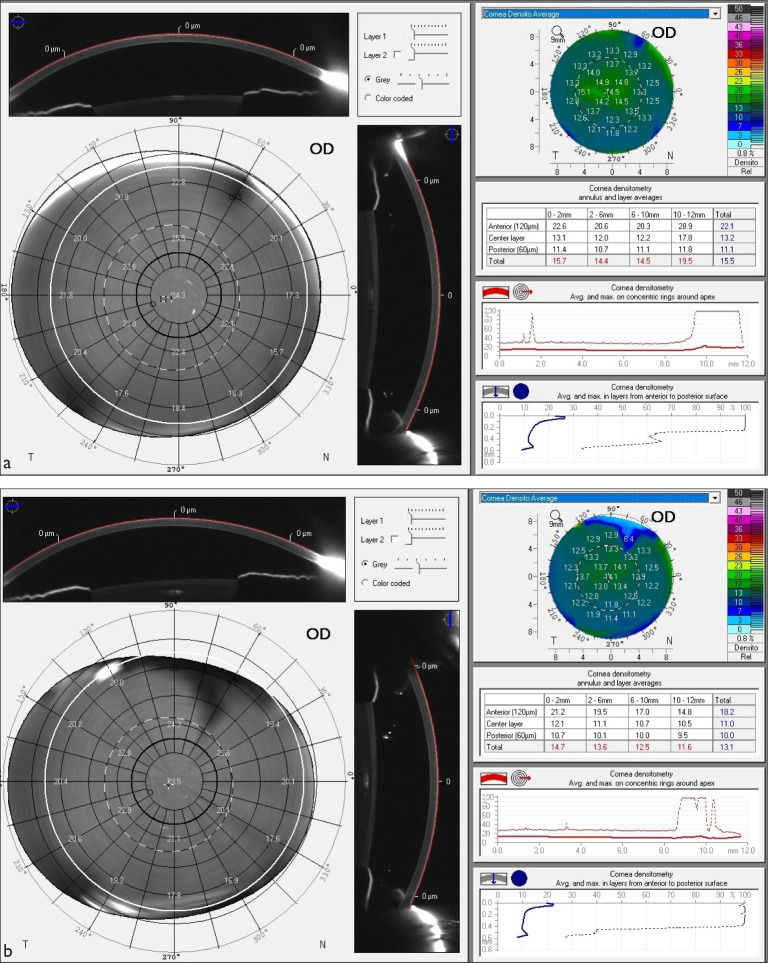
**(a)** Preoperative corneal densitometry images of a patient with chalazion. **(b)** Corneal densitometry images of a patient with chalazion at first month postoperatively.

Corneal densitometry values in the anterior region were 21.71±3.08 in Group 1, 19.98±3.73 in Group 2, and 19.03±1.59 in Group 3 (Groups 1–2: p=0.039, Groups 1–3: p≤0.001, and Groups 2–3: p=0.478). Corneal densitometry values in the central region were 13.40±1.70 in Group 1, 12.95±2.00 in Group 2, and 12.24±1.33 in Group 3 (Groups 1–2: p=0.773, Groups 1–3: p=0.01, and Groups 2–3: p=0.213). In the posterior region, it was 11.29±1.23 in Group 1, 10.98±1.19 in Group 2, and 10.62±1.20 in Group 3, and the difference was not significant (p=0.055). When the total corneal densitometric measurements were evaluated, it was 15.95±1.80 in Group 1, 14.76±1.76 in Group 2, and 14.33±1.49 in Group 3 (Groups 1–2: p=0.01, Groups 1–3: p≤0.001, and Groups 2–3: P = 0.804). Chalazion patients had higher densitometry values compared to the healthy group. Besides, a statistically significant decrease was observed in the anterior (10–12 mm), central (6–10 mm), and total corneal densitometry values in the post-operative period (Table 2). Pre- and post-operative corneal densitometry images of a patient with chalazion are shown in Figure 1a and b.

**Table 2.** The comparison of corneal densitometry values of preoperative chalazion, postoperative chalazion, and healthy control group

	Group 1	Group 2	Group 3	p*	p^†^
	(Preoperative)	(Postoperative)	(Control)		
	(n=36)	(n=36)	(n=40)		
Anterior (120μm) (GSUs)					
0-2 mm	20.88±1.38	20.35±1.04	19.77±0.89	**<0.001**	**1-3: <0.001**
2-6 mm	19.64±1.30	19.23±0.60	18.79±1.62	**0.015**	**1-3: 0.011**
6-10 mm	18.91±1.82	18.09±0.87	17.59±1.85	**0.002**	**1-3: 0.001**
10-12 mm	27.47±3.55	23.39±2.87	21.95±3.59	**<0.001**	**1-2: 0.012**
					**1-3: <0.001**
Total	21.71±3.08	19.98±3.73	19.03±1.59	**<0.001**	1-2: 0.039
					**1-3: <0.001**
					2-3: 0.478
Central (GSUs)					
0-2 mm	13.00±0.81	12.68±0.89	12.45±0.71	**0.013**	**1-3: 0.001**
2-6 mm	11.72±0.62	11.46±0.58	11.30±0.60	**0.010**	**1-3: 0.008**
6-10 mm	12.50±1.56	11.71±1.04	11.13±0.84	**<0.001**	**1-2: 0.016**
					**1-3: <0.001**
10-12 mm	17.86±3.87	15.94±2.39	15.12±2.93	**0.001**	**1-3: 0.001**
Total	13.40±1.70	12.95±2.00	12.24±1.33	**0.012**	1-2: 0.773
					**1-3: 0.010**
					2-3: 0.213
Posterior (60μm) (GSUs)					
0-2 mm	10.97±0.87	10.81±0.74	10.59±0.68	0.100
2-6 mm	10.34±0.65	10.14±0.42	9.94±0.69	**0.017**	**1-3: 0.013**
6-10 mm	11.46±1.26	11.10±1.69	10.41±0.79	**0.002**	**1-3: 0.002**
10-12 mm	13.95±2.92	13.11±2.94	12.21±1.98	**0.018**	**1-3: 0.014**
Total	11.29±1.23	10.98±1.19	10.62±1.20	0.055	1-2: 0.800
					1-3: 0.049
					2-3: 0.596
Total (GSUs)					
0-2 mm	15.01±0.96	14.73±1.14	14.35±1.26	**0.043**	**1-2:0.039**
					1-3:0.873
					2-3: 0.458
2-6 mm	13.62±0.86	13.33±0.91	12.94±1.04	**0.008**	**1-3: 0.006**
6-10 mm	14.25±3.00	12.95±1.10	12.40±0.90	**<0.001**	**1-2: 0.013**
					**1-3: <0.001**
10-12 mm	20.74±2.58	18.74±3.89	17.43±3.00	**0.009**	**1-3: 0.007**
Total	15.95±1.80	14.76±1.76	14.33±1.49	**<0.001**	**1-2: 0.010**
					**1-3: <0.001**
					2-3: 0.804

Results are denoted as mean±standard deviation. GSU: Gray Scale Units. *: One-way analysis of variance (ANOVA); p<0.05 statistically significant. ^†^: Bonferroni Post-hoc test; p<0.016 statistically significant. (Bold value indicates statistically significant).

In corneal aberration, measurements made according to site of chalazion, while there was a statistical difference in total RMS measurements (p=0.035), there was no difference in spherical aberration, trefoil horizontal, trefoil oblique, coma horizontal, and coma vertical values (p=0.192, p=0.283, p=0.067, p=0.168, and p=0.087; respectively) (Table 3).

**Table 3.** Corneal aberration measurements according to site of chalazion

	Upper eyelid group	Lower eyelid group	p*
	(n=20)	(n=16)	
Total RMS (μm)	1.79±0.42	1.45±0.48	**0.035**
Spherical Aberration (μm)	0.172±0.064	0.197±0.045	0.192
Trefoil Horizontal (μm)	0.079±0.086	0.035±0.138	0.283
Trefoil Oblique (μm)	-0.001±0.086	-0.069±0.120	0.067
Coma Horizontal (μm)	0.077±0.173	0.012±0.098	0.168
Coma Vertical (μm)	0.172±0.064	0.064±0.205	0.087

RMS: Root mean square μm: micron meters. Results are denoted as mean±standard deviation. *: Independent t-test; p<0.05 statistically significant. (Bold value indicates statistically significant).

When the corneal densitometry values of the patients with chalazion in the upper and lower eyelids were compared, no statistically difference was found in the central (p=0.08) and posterior region (p=0.542), while the anterior and total corneal densitometry values were higher in patients with chalazion in the upper eyelid, and the difference was statistically significant (p=0.039 and p=0.032; respectively) (Table 4).

**Table 4.** Corneal densitometry values according to site of chalazion

	Upper eyelid group	Lower eyelid group	p*
	(n=20)	(n=16)	
Anterior (120μm) (GSUs)			
0-2 mm	21.06±1.47	20.65±1.28	0.378
2-6 mm	19.81±1.48	19.44±1.04	0.398
6-10 mm	19.48±1.97	18.20±1.37	**0.028**
10-12 mm	26.50±1.12	24.53±2.77	**0.015**
Total	22.00±1.45	21.06±1.19	**0.039**
Central (GSUs)				
0-2 mm	13.03±0.85	12.96±0.78	0.807
2-6 mm	11.74±0.72	11.70±0.50	0.864
6-10 mm	12.69±1.98	12.27±0.77	0.391
10-12 mm	18.12±5.03	16.77±1.33	0.263
Total	13.81±2.16	12.89±0.55	0.080
Posterior (60μm) (GSUs)			
0-2 mm	10.86±0.72	11.11±1.03	0.405
2-6 mm	10.30±0.62	10.39±0.70	0.677
6-10 mm	11.58±1.60	11.31±0.64	0.508
10-12 mm	13.72±3.75	14.25±1.41	0.561
Total	11.19±1.50	11.43±0.78	0.542
Total (GSUs)			
0-2 mm	15.06±1.01	14.95±0.91	0.746
2-6 mm	13.68±1.02	13.55±0.63	0.643
6-10 mm	14.80±3.92	13.58±0.80	0.190
10-12 mm	21.62±5.90	19.88±1.59	0.221
Total	16.27±1.73	15.28±0.74	**0.032**

Results are denoted as mean ± standard deviation. GSU: Gray Scale Units. *: Independent t-test; p<0.05 statistically significant. (Bold value indicates statistically significant).

## Discussion

The etiopathogenesis in chalazion formation remains uncertain. The host inflammatory response against the infectious agent occurs when the infectious agent and/or its metabolites activate the humoral and cellular response (6). It is known that some preparative factors that cause chalazion formation in chalazion patients may cause dry eye formation. The Schirmer I test and corneal fluorescein staining are frequently used to determine the presence and severity of dry eyes. In dry eye, confocal biomicroscopy has shown an increase in the density of inflammatory cells in the corneal epithelium and hyperreflective keratocytes, possibly induced by inflammatory mediators (7, 8). It has also been reported that dry eye may affect optical visual quality by changing optical aberrations (9). Researchers have reported that the presence of punctate epitheliopathy in patients with dry eyes further increases the corneal densitometry value (10). However, Fukuoka et al. (11) reported that there was a decrease in the Schirmer test in chalazion patients compared to the healthy control group, but this difference was not significant. Chalazion patients with a Schirmer I test score <10 mm and/or punctate staining on the cornea were excluded in the current study because it might affect the corneal densitometry measurements. However, even in this case, Schirmer I test values were lower than in the control group. This may be associated with the concomitant presence of meibomian gland dysfunction, ocular surface disorder, chronic blepharitis, tear instability, and complex inflammatory processes in chalazion patients, which are all involved in the etiopathogenesis of dry eye.

Not all regions of the cornea have the same biomechanical properties. Whereas the collagen in the cornea is arranged in an inferior-superior and nasal-temporal manner, it shows a tangential course in the limbus (12). In addition, elastic differences in the cornea are less in the cornea paracentral and periphery but greatest in the limbus (13). This situation causes the cornea to respond differently to mechanical effects. In several previous studies, it was revealed that the chalazion resulted in changes in corneal astigmatism and aberrations due to the effect of mechanical compression (14-16). In addition, it has been reported that chalazion may cause an increase in intraocular pressure and that a decrease in intraocular pressure would be achieved after excision (17). It has been stated that lesions on the upper eyelid, involving the central eyelid and that are >5 mm, have a greater effect on corneal astigmatism and aberrations. Therefore, early excision of these lesions is recommended (14-16).

Corneal aberrations are related to the image quality of the retina. Increases in ocular aberrations result in a decrease in optical visual quality due to the development of glare, halo, and distortion (18). It is known that the increased eyelid pressure caused by the lesion effect in the chalazion increases HOAs (16). Sabermoghaddam et al. (16) reported a decrease in ocular aberrations after excision in patients with upper eyelid chalazion. Jin et al. (15) reported that vertical astigmatism, oblique astigmatism, and total RMS aberrations were higher in the upper eyelid compared to lesions in the lower eyelid. In the present study, it was observed that total RMS, spherical aberration, and trefoil horizontal aberration values were higher in patients with chalazion compared to the healthy control group. In these patients, there was a significant decrease in total RMS and coma vertical aberrations after surgery. In the studies mentioned above, only patients with chalazion in the upper eyelid were evaluated. However, in the present study, aberrometric evaluation was performed in patients with chalazion in the lower and upper eyelids. Total RMS values were found to be higher in patients with chalazion in the upper eyelid. Considering the effects of changes in corneal aberrations on optic quality, especially upper eyelid lesions, should be excised at an early stage.

Many studies have reported that there may be changes in corneal keratometry and refraction in patients with chalazion. However, it is known that these changes do not affect corneal densitometry measurements (19, 20). Therefore, refractive and keratometric changes observed in the chalazion are not seen as a confounding factor in corneal densitometry measurement in this study.

The Pentacam Scheimpflug system is a superior imaging method in many aspects than light biomicroscopy in detecting corneal pathologies. Corneal densitometric measurements are made with the addition of a new software to the Pentacam HR device; thus, objective, fast, and reproducible data are obtained (21, 22). With this imaging method, possible densitometric changes can be detected even in corneas that appear completely transparent clinically. A healthy cornea does not normally absorb visible light, so the light distribution is minimal (3). However, proteoglycans surrounding the keratocyte and collagen fibrils in the cornea and disorders in the extracellular matrix organization may decrease vision quality due to increased light backscatter (23). The increases in corneal density are not necessarily related to the decrease in vision; such increases are thought to be related to decrease visual quality (24).

There is still no consensus on what constitutes normal values in corneal densitometry. Whereas the total corneal densitometry value was measured as 19.74±3.89 in a study of 445 healthy participants (5), it was measured as 14.4±2.74 in another study with 588 participants (19). In the present study, the total corneal densitometry value was 15.95±1.80 in chalazion patients and 14.33±1.49 in the healthy control group. It was observed that corneal densitometry values were 14.76±1.76 in the post-operative 1^st^ month, and the decrease in these patients after surgical excision was significant. Furthermore, results revealed that corneal densitometric changes in the chalazion occurred more frequently in the anterior and central areas compared to the posterior area. This result may relate to the lower density of keratocytes in the posterior layer.

One or more mechanisms may have caused the increase in corneal densitometry in patients with chalazion. Normal cornea distributes light predominantly at the air-tear film and tear film corneal interface; therefore, the change in the refractive index of light in the anterior is highest (25). The inflammatory process in chalazion, mechanical irritation on the ocular surface, conjunctival inflammation, and the pre-ocular tear film layer imbalance may have caused an increase in densitometry values. Tanaka et al. (26) stated that conjunctival inflammation might lead to the development of corneal damage. In the chalazion, a conjunctival inflammatory process occurs against the infectious agent, its toxins and mediators, and this process is accompanied by a host response in the limbus. Subclinical keratocyte activation develops through the activation of pro-inflammatory cytokines and matrix metalloproteinase-like enzymes (3). This complex inflammatory process may trigger remodeling in the corneal stroma, leading to an increase in corneal densitometry. In addition, medical treatment applied after chalazion excision in these patients may have contributed to the reduction of corneal densitometry values by eliminating infectious agents and inflammatory mediators and causing a decrease in conjunctival inflammation. However, the reason for corneal densitometry values not returning to normal after surgical excision remains unclear. These results are pre-sumambly related to the short follow-up period. In studies performed in patients undergoing refractive surgery, keratocyte activation that can persist for months after the intervention has been reported (27,28). In addition, the researchers noted that the high corneal densitometry values observed in the keratitis areas during the active infection period were still higher than normal, although the keratitis area appeared to be completely healed on biomicroscopic examination (29). The present study observed that corneal densitometry values decreased in the post-operative period and were similar to the values in the healthy control group. In addition, it was observed that corneal densitometry values were higher in anterior and total regions of the upper eyelid compared to lesions in the lower eyelid. This may be related to the biomechanical properties of the cornea, changes in the corneal structure in patients with chalazion, or more contact of the chalazion in the upper eyelid with the cornea due to gravity. This result, in addition to aberrometric changes in the cornea, may be another reason for the early excision of the upper eyelid chalazion.

This study had some limitations. First, a small number of patients included in this study. This result needs to be supported by a larger number of patients. Second, in vivo confocal microscopy was not provided to the participants to support these findings. Possible microstructural changes in the cornea in chalazion should be supported by in vivo confocal microscopic studies. Finally, in the present study, the patients were evaluated in the 1^st^ post-operative month. Long-term results after excision should also be observed in these patients.

## Conclusion

It was observed that some corneal aberration values and corneal densitometry values were higher in chalazion patients than in a healthy control group, and there was a decrease in corneal aberrations and densitometry values after surgical excision in this study. Although improvement was observed in densitometric and aberrometric values in the early period after excision, problems in optical quality may persist unless the predisposing factors causing chalazion development are completely eliminated.

## Disclosures

### Ethics Committee Approval:

Diyarbakır Gazi Yaşargil Training and Research Hospital (08.11.2019/366).

### Peer-review:

Externally peer-reviewed.

## Conflict of Interest:

None declared.

## Authorship Contributions:

Involved in design and conduct of the study (HO, UD, MFA, YY); preparation and review of the study (HO, YY, MC); data collection (HO, UD, MFA); and statistical analysis (MC, HO).
